# Reaction Process of Solid Waste Composite-Based Cementitious Materials for Immobilizing and Characterizing Heavy Metals in Lead and Zinc Tailings: Based on XRD, SEM-EDS and Compressive Strength Characterization

**DOI:** 10.3390/molecules29050996

**Published:** 2024-02-25

**Authors:** Jianwei Lu, Dun Wu, Shuqin Li, Xia Gao

**Affiliations:** 1 Anhui Province Intelligent Underground Exploration and Environmental Geotechnical Engineering Research Center, State Key Laboratory of Safety and Health for Metal Mines, College of Civil Engineering, Anhui Jianzhu University, Hefei 230601, China; lu1245076898@163.com; 2 School of Earth and Space Sciences, University of Science and Technology of China, Hefei 230026, China; 3 Sinosteel Maanshan General Institute of Mining Research Co., Ltd., Maanshan 243000, China; lishuqin03@163.com; 4 School of Architecture and Urban Planning, Anhui Jianzhu University, Hefei 230601, China; gaoxia@ahjzu.edu.cn

**Keywords:** lead and zinc tailings, solid waste-based gelling materials, gelling activity, synergy, immobilization mechanism

## Abstract

This study investigates the synergistic effect and mechanism of gelling materials with blast furnace slag (BFS), steel slag (SS) and desulphurization gypsum (DG) as the main components on the hardening of heavy metal ions by lead and zinc tailings. It is found that lead and zinc tailing (LZT) is mainly composed of dolomite and quartz and contain small amounts of calcium, aluminum, iron, magnesium and other elements as well as heavy metals such as lead and zinc. By the mechanical activation method, it is found that the lead and zinc tailings powder has the largest specific surface area and the highest activity index when the ball milling time is 2 h. At a hardening timepoint of 28 d, the calcite crystals in the samples are intertwined with the amorphous C-S-H gel (C-S-H gels are mainly composed of 3CaO∙SiO_2_ and 2CaO∙SiO_2_), which enhances the structural strength of the samples. The chemical reaction analysis confirmed that the formation of calcite is a major driver for the hydration reaction of the steel slag–desulphurization gypsum (SSSDG) system. Overall, the slag, steel slag and desulphurization gypsum solid waste-based gelling materials have synergistic effects in hardening heavy metals by limiting the leaching of metal ions, adsorbing metal ions and hardening heavy metals, and facilitating the hydration process through the formation of compound salt precipitates.

## 1. Introduction

The use of heavy metal-containing materials in infrastructure is an issue that needs to be handled with care, mainly because of the potential long-term negative impacts of heavy metals on the environment and human health. While there may be a need for the use of heavy metal-containing materials in certain infrastructure applications, in view of their potential environmental and health risks, their use should be strictly controlled, and the use of safer and more environmentally friendly alternative materials and technologies should be actively sought and promoted. LZT is a type of non-ferrous metal tailings, and China is the world’s largest producer and consumer of non-ferrous metals. In 2022, China’s refined zinc production and consumption accounted for 43.10% and 47.50% of the world’s total, and the country’s refined lead production and consumption accounted for 41.50% and 42.30% of the world’s total [1]. However, China’s lead and zinc ores are mostly poor and of low grade, and it takes a complicated beneficiation process to obtain the concentrate, resulting in a large number of tailings being discharged. The continuous accumulation of a large number of undeveloped LZT has had a great impact on the environment. On the one hand, the accumulation of tailings has occupied and destroyed a large amount of land resources, restricted the development and utilization of land, and reduced the arable land area of the country. The end result of this has been to limit the development of agriculture [2,3,4]. On the other hand, tailings ponds have high energy potential. It is easy for them to cause safety hazards and a risk of collapse, once they are broken down, and it is very easy for them to block the valley to cause flash floods, causing great disasters and losses to downstream industrial and agricultural production as well as to people’s lives and property; at the same time, the tailings contain residual toxic elements such as lead, zinc, cadmium, etc., and are prone to wind propagation and water erosion. If not properly managed, the leaching of these hazardous substances inevitably endangers the environment and poses a serious threat to public health [5,6].

In recent years, the tailings disposal method and comprehensive environmental management have been highly appreciated by our government departments. The construction of tailings ponds, in many places, to deal with tailings still cannot avoid environmental pollution, but the cementing and filling of tailings in empty areas and the tailings’ heavy metal immobilization and stabilization disposal are an important means to doing so [7]. At present, heavy metal pollution prevention and control technology mainly includes soil leaching technology, electrokinetic remediation, bioremediation, thermal desorption, immobilization stabilization technology, vitrification technology, cement kiln co-disposal technology, the guest soil method and so on. Du et al. (2022) [8] employed the iron tailings from two tailing ponds in a mining operation in Tibet as a research object and used the improved BCR method to investigate the morphological distribution of nine heavy metals in iron tailings. BCR extraction is a technique used to analyze the forms of heavy metals in soils or sediments. This is carried out by a sequential extraction process that classifies the heavy metals into different chemical forms, including the exchangeable state, the reduced state (ferromanganese oxide bound), the oxidized state (organic and sulfide bound) and the residual state. The BCR extraction method is a valuable tool for investigating the behavior of heavy metals in environmental media. It provides a detailed distribution of heavy metal forms and assists in environmental risk assessments. It also provides a scientific basis for environmental management and policy formulation. The results of Du et al.’s study showed that Pb^2+^, Cu^2+^, Cd^2+^ and Zn^2+^ in the iron tailings were in more than 60% of the easily migratory state, which had potential risks for the environment, among which Pb was mainly in the reduced state, and Cu^2+^, Cd^2+^ and Zn^2+^ were mainly in the oxidized state. Wei et al. (2022) [9] conducted simulation tests of heavy metal leaching on tailings with different particle sizes using both static leaching and dynamic leaching to analyze the changes in leaching concentrations of heavy metals (Cu^2+^, Mn^2+^, Zn^2+^) in tailings samples. The results indicated that the leached concentration of heavy metals under dynamic leaching showed an obvious decreasing trend with an increase in the leaching amount, and there were two stages of rapid and slow decrease. Guo et al. (2022) [10] established an evaluation index system for remediation variables using the mechanism and law of “electric remediation + phytoextraction” method. They integrated the random forest classifier and GRA simulation algorithm to establish the soil simulation remediation GRA-RF model. The results show that the simulated remediation efficiency of the eight remediation samples is higher than the experimental remediation efficiency. Zhao et al. (2023) [11] investigated the effect of cement, “cement + phosphorus-based agent A” and “cement + organic chelating agent B” as immobilizing/stabilizing agents to solidify/stabilize heavy metals in lead and zinc waste rock tailings, respectively. The results of the pharmaceutical dosing program were 5% to 10% cement + 1% to 2% phosphorus-based agent. A. Fang et al. (2021) [12] measured the properties of the improved substrate and the physiological and ecological characteristics of plants, as well as the heavy metal content under the condition of tailings soil mixing improvement. The study found that soil improvement could effectively enhance the physicochemical properties of copper tailings, promote plant growth, and alleviate heavy metal stress. The test was conducted following the principle of cement immobilization, using a new type of cementitious material for the immobilization and stabilization of heavy metal tailings. The study utilized industrial solid wastes, including BFS, SS, DG, and other similar materials, as the primary raw materials to produce a cost-effective, high-performance cementitious material. The resulting material exhibited excellent characteristics for immobilizing and stabilizing heavy metals. A mechanical activation test was conducted on LZT cementum for a mine in Maanshan, Anhui Province, to investigate its potential impact on the activity of LZT samples. The purpose was to explore the synergistic effect and mechanism of this gelling material for immobilizing heavy metal tailings [13].

This paper proposes a method for the comprehensive utilization of industrial solid waste to prepare cementitious materials for curing heavy metal tailings. The main raw materials used for the preparation of cementitious materials were BFS, SS and DG industrial solid wastes, which reduced the cost and improved the utilization rate of resources. And, for the harmless and resourceful utilization of LZT, a new type of gelling material was adopted for curing and stabilization, which was of important environmental significance. Secondly, the effect of ball milling time on the particle size distribution and activity index of LZT powder was studied via the mechanical activation method, and the milling process was optimized. It provided a reliable scientific basis and data support for the harmless and resourceful utilization of LZT in the future, which was innovative and had practical value.

## 2. Results and Discussion

### 2.1. Physical and Chemical Characterization of LZT

**Figure 1:**
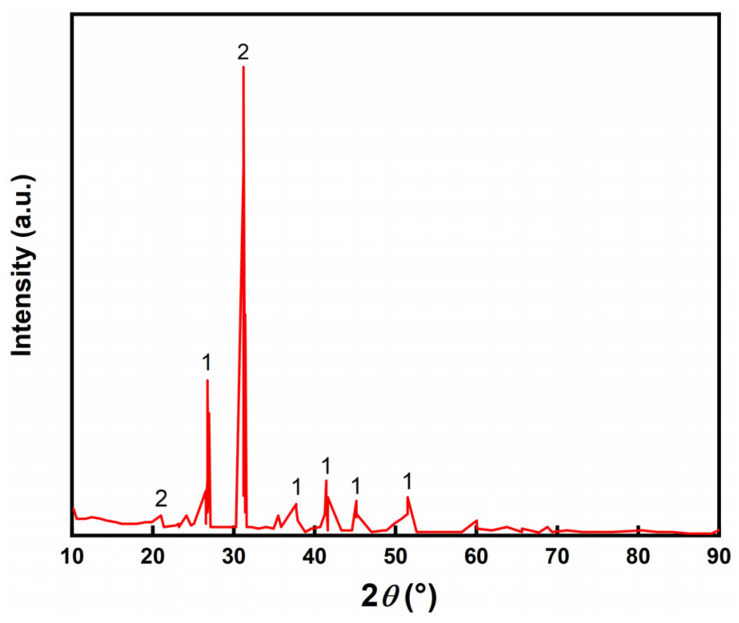
XRD pattern of LZT (1—dolomite; 2—quartz).

The XRD pattern of LZT is shown in Figure 1, from which it can be seen that the main physical phases of LZT were dolomite and quartz. Table 1 shows the analysis of XRD patterns. From Table 1, the chemical composition of the LZT test results can be observed. Its main chemical composition consists of silicon (Si), calcium (Ca), aluminum (Al), iron (Fe), magnesium (Mg) and other elements, and it also contains a small amount of lead (Pb) and zinc (Zn).

### 2.2. Effect of Ball Milling Time on Particle Size Distribution of LZT Micropowder

**Figure 2:**
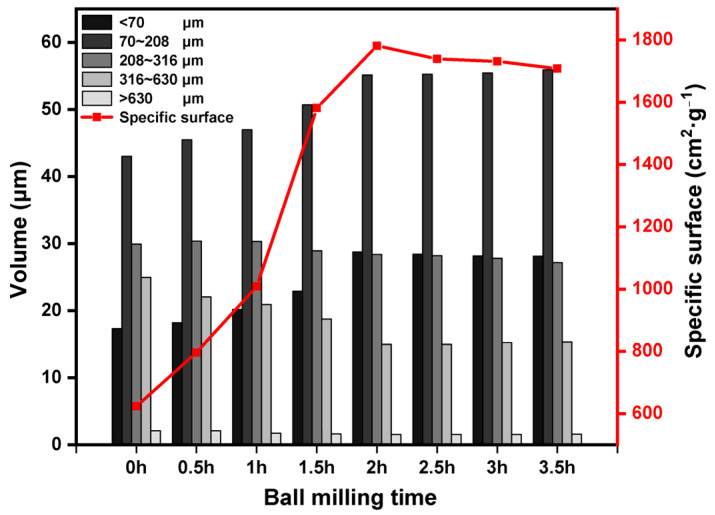
Particle size distribution and specific surface area of LZT micropowder with different ball milling time.

As can be seen in Figure 2, the particles of the LZT micropowder were refined by the process of mechanical force ball milling, and the percentage of particle size less than 70 μm increased from 17.32% to 28.74% of the original tailings. With the extension of ball milling time from 0.5 h to 2 h, the specific surface area of the LZT micropowder increased from 796.12 cm^2^/g to 1781.40 cm^2^/g. However, with the continued extension of the milling time, there was little change in the specific surface area, and the material appeared to be agglomerated, and the particle size increased instead. Therefore, the optimized grinding time for mechanical force activation was 2 h, at which time the specific surface area of the LZT micropowder was 1781.40 cm^2^/g.

### 2.3. Comparative Analysis of the Physical Properties of LZT Micropowder before and after Ball Milling

**Figure 3:**
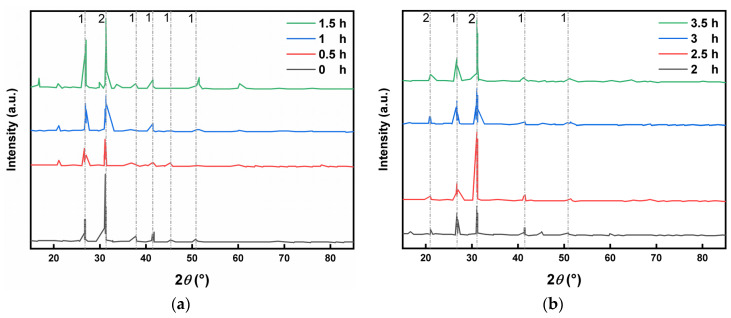
XRD patterns of LZT micropowder at different grinding times (1—dolomite; 2—quartz). (**a**,**b**) XRD characterisation of the mineral fraction of LZT micropowder before and after ball milling.

As shown in Figure 3a, the main were dolomite and quartz, and the diffraction peaks of quartz in the unground the LZT micropowder were fewer and weaker, and the diffraction peaks of quartz (*100*) and (*001*) crystal planes were observed only at 2*θ* angles of 31°. At the early stage of mechanical milling, the relative intensities of the (100) and (*001*) crystal plane diffraction peaks of quartz increased with the prolongation of the mechanical milling time, and the diffraction peaks corresponding to the (*110*), (*102*), (*112*), and (*121*) crystal planes appeared at 36.55°, 39.47°, 50.14°, and 59.96°, respectively, with a gradual increase in the specific surface area of the tailings micropowder. Huang et al. (2021) [14] concluded that with the extension of the grinding time, the harder quartz was ground, the mineral particles became finer, the scattering was enhanced, and the diffraction peaks increased. Furthermore, as the grinding time increased, the greater the damage, the more severe the lattice deformation and the weaker the diffraction peak. This was fully consistent with the experimental results which showed that since the hardness of quartz is about 7 and that of dolomite is about 3.5, an increase in the specific surface area of quartz and dolomite after crushing with an increase in the grinding time led to an increase in their diffraction peaks. Observing Figure 3b, the diffraction peaks of quartz and dolomite showed a tendency to increase and then decrease in mechanical milling. This may be due to the fact that the crystalline phase was revealed by the initial milling, after which the crystal structure of the minerals was destroyed due to excessive milling, and some of the minerals turned into an amorphous phase.

### 2.4. Effect of Ball Milling Time on the Activity Index of LZT Micropowder

**Figure 4:**
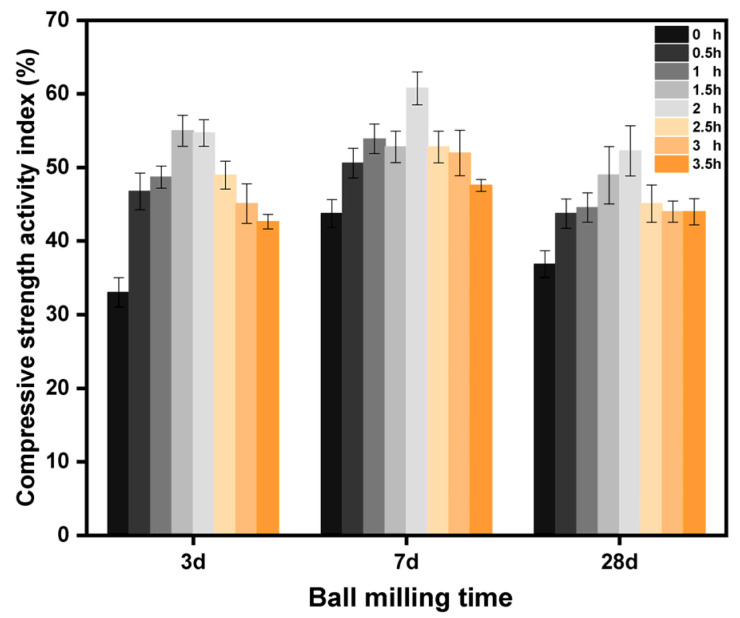
Activity of LZT at different ball milling times.

Table 2 shows the compressive strength of cemented sand specimens at 3 d, 7 d, and 28 d, obtained by mixing the LZT micropowder with benchmark cement in the same proportion and varying the ball milling time. Figure 4 illustrates the activity of the LZT micropowder at different ball milling times. Table 2 shows that the compressive strength of the composite system increased and then decreased with the increase in ball milling time. The early compressive strength increase was more obvious with the maintenance age growth. The change in the later 28 d compressive strength and 7 d compressive strength was not significant. The change rule in Figure 4 for the activity index was also the same. The finer particles and increased specific surface area resulting from ball milling LZT improved the hydration rate of the cement, allowing for a sufficient hydration reaction and ultimately increasing the compressive strength of the test pieces. However, when the ball milling time exceeds 2 h, the specific surface area increases, leading to a higher water requirement for the composite system. The remaining water evaporates after the cement hydration reaction, leaving behind numerous pores inside the specimen, which results in lower strength. Finally, as could be seen from Table 2, the COV gradually decreases with the increase in the number of days of curing. This signified that the compressive strength of the cured specimens became more stable and consistent. It also reflected that the stability and reliability of the compressive strength of the cured specimens increased.

### 2.5. Study on the Synergistic Mechanism of SSSDG Solid Waste-Based Gelling Agent under Chemical Excitation

The mineralogical composition of SS was similar to that of cement clinker, and the aqueous interpretation of SS releases OH^-^, but the activity of SS was much lower than that of the clinker, which resulted in a much lower rate of OH^-^ release than that of the clinker. Preetham H et al. (2019) [15] concluded that OH^-^ breaks the chemical bond between SiO_2_-CaO-Al_2_O_3_. This coincides with the fact that the dissolution of BFS releases activated silicate ions as well as Ca^2+^, Mg^2+^, and Al^3+^, which react with OH^-^ to form gels such as C-S-H and C-A-H. This process required SS to stimulate the activity of BFS, so that the BFS hydration could take place. S hydration consumed OH^-^ to improve the rate of dissolution and the hydration of SS, SS and BFS hydration reactions, the mutual assistance and mutual promotion of SS and BFS hydration, the generation of a large number of C-S-H as well as C-A-H (C-A-H usually refers to hydrated calcium aluminum silicate, a major hydration product in cementitious materials) and other gels. The cementitious material contained a significant amount of gypsum. Excess gypsum reacted with the C-A-H gel, producing needle-and-rod AFT (calcite), which was consumed to produce calcite. According to the principle of equilibrium in chemical reactions, the consumption of gel promotes the hydration of SS and BFS, which are complementary and mutually reinforcing. This process accelerated the dissolution of SS and BFS while promoting the hydration reaction, resulting in the formation of ettringite [16], gel, and various metal ions, such as hydrated silicate hydration products.

The hydration process of cementitious materials is as follows:

*n*SiO_2_∙*m*Al_2_O_3_∙*q*CaO + *r*Ca(OH)_2_ + *s*H_2_O = *n*SiO_2_∙*m*Al_2_O_3_∙(*q* + *r*)CaO∙(*s* + *r*)H_2_O

Slag aluminum-containing C-S-H gels

*n*SiO_2_∙*m*Al_2_O_3_∙*q*CaO + *r*Ca(OH)_2_ + *s*H_2_O + *3m*CaSO_4_∙2H_2_O

Slag gypsum

=*n*SiO_2_∙(*q* + *r* − *3m*)CaO∙(*s* + *r* − *27m*)H_2_O + *m*(3CaO∙Al_2_O_3_∙CaSO_4_∙32H_2_O)

C-S-H gels ettringite

In order to further validate the analysis to obtain the conclusion of hydration products and the hydration reaction process, SEM was used to observe and compare the fabricated SS-based cementitious materials.

**Figure 5:**
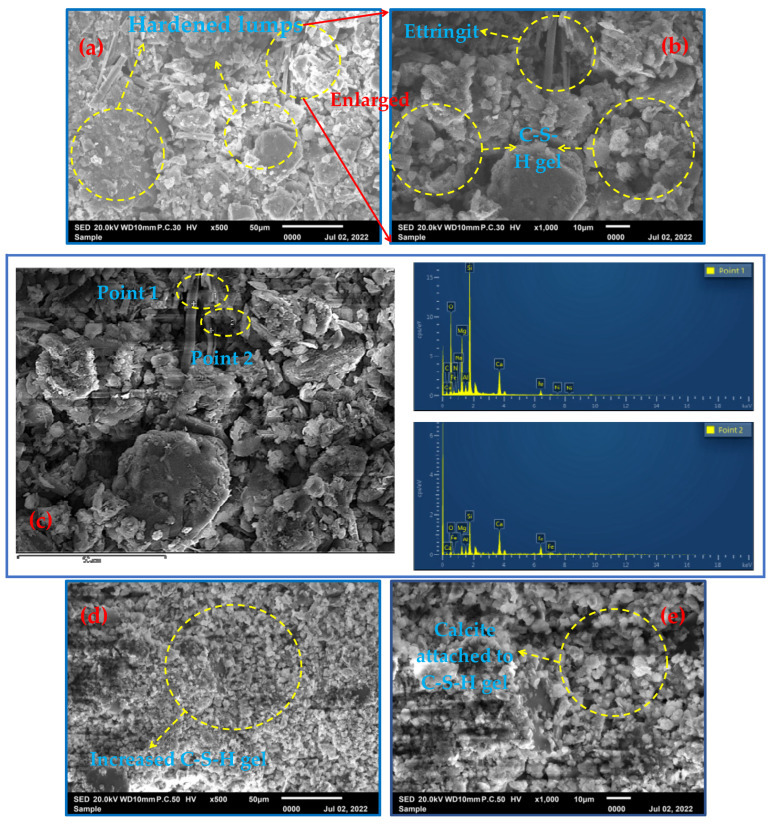

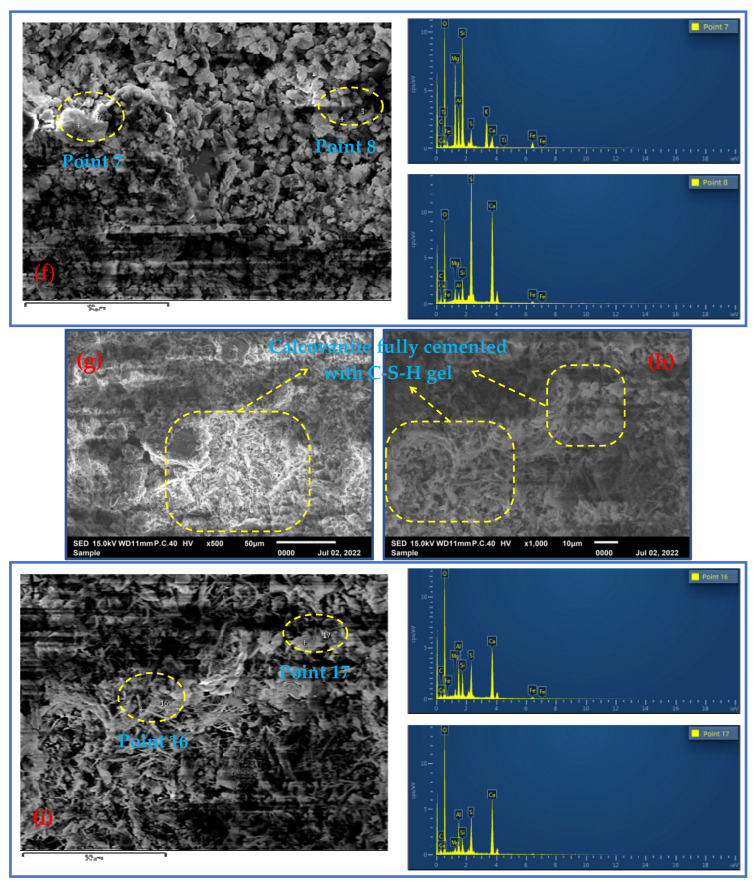
The SEM-EDS spectra at different conservation time (3 d, 7 d and 28 d). (**a**) shows a scanning electron microscope (SEM) image of the reaction product that appears to have hardened after 3 days of the filling slurry. A hydrated calcium silicate gel (C-S-H gel) is visible in (**b**), with a magnification of 1000. (**c**) is a local magnification of (**b**), and the elemental compositions of the two probes are given by energy-dispersive X-ray spectroscopy (EDS). (**d**) shows an SEM image of the 7-day specimen, with a magnification of 500. Calcite is attached to the C-S-H gel on calcite at a magnification of 1000. (**f**) is a local enlargement of (**e**) and displays the elemental composition of the two probes using EDS. (**g**) and (**h**) show the SEM microstructure of the specimen at 28 days. (**i**) is a local enlargement of (**h**) and displays the elemental composition of the two probes using EDS.

**Figure 6:**
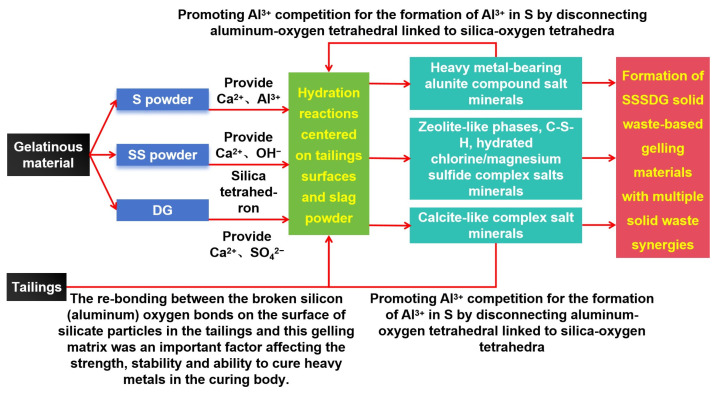
Role of various solid wastes in gelling material systems.

Figure 5a shows that the reaction products appeared and began to harden at 3 d of the fill slurry, with primary minerals accumulating and filling in between the whole tailings sand particles, cementing and adhering the whole sand particles and showing early strength. This was due to the formation of a small amount of needle-shaped material (calcovanadite). Figure 5b shows that although the production of calcovanadite favored the early strength of the filler, magnification revealed that the calcite crystals were small in diameter, incomplete and in the early stages of growth, while a small amount of amorphous C-S-H gel was seen. From Figure 5c, which shows the energy spectra of point scans 1 and 2, it can be seen that the filled slurry was mostly in the primary mineral phase (cordierite suspect, dolomite, etc.), with only a small amount of fibrous material (calcite) produced, and the bands and lumps were basically in the primary mineral phase. Figure 6d,e present the SEM microstructures of the 7-day specimen at the point of conservation, the latter being a magnified view. Additionally, Figure 5d shows that the amorphous C-S-H gel observed at 7 days also gradually increased. Furthermore, Figure 5e shows that by 7 days of maintenance, the calcovanadite crystal content increased, attached to the C-S-H gel, and intertwined with calcovanadite and all-tailed sand particles. The overall structure of the gel appears to be more compact at the point of 7 days. EDS confirmed the presence of primary minerals, including cordierite (as shown in spot scan 7), while calcovanillite attached to the C-S-H is generated, as shown in the results of spot scan 8. The SEM microstructure of the 28-day specimen is illustrated in Figure 5g and 5h, and the formation of calcovanadite was much larger in diameter and tightly and densely connected. The specimen contained interwoven calcovanite and amorphous C-S-H gel, which were closely connected to the tailing sand particles, creating a structure similar to reinforced concrete. The EDS analysis from Figure 5i showed the presence of a significant cross-network structure with a large number of C-S-H phases and needles/filaments (calcovanite), as well as a pronounced gel structure. Additionally, the analysis directly demonstrated the presence of calcite (spot scan 16) and C-S-H (spot scan 17).

From the above analysis, it was easy to see that the generation of calcite was a great driving force for the hydration reaction of the SSSDG system. As studied by Özkök E et al. (2019) [17], under alkaline conditions, calcite was an extremely low-solubility complicated salt with a solubility product constant of 10^−111.6^, and the calcite complicated salt formed needle and rod crystals with nanometer diameters, which had a strengthening and toughening effect on the hardened body. In the SSSDG system, BFS was rich in calcium–silicon and aluminum elements, and the converter slag was rich in calcium–silicon and divalent metal elements, and the three main raw materials could play their respective roles in the system.

Wang et al. (2021) [18] demonstrated that depolymerizing silica-oxygen tetrahedra and aluminum–oxygen tetrahedra generated numerous reactive silica-oxygen tetrahedra in the system. These tetrahedra combined with Ca^2+^ ions in the liquid phase, forming more and more C-S-H gels, either as single silicate ions or as polymers of multiple silicate ions. Rodriguez-Navarro et al. (2015) [19] revealed that the formation of calcite necessitates a constant supply of Ca^2+^, OH^−^, and SO_4_^2−^ ions and divalent metal cations. These ions drive the reaction to continue and form a large number of calcite crystals that are extremely difficult to dissolve. Hou et al. (2023) [20] discovered that the average distance between silica-oxygen bonds is 0.160 nm, which is smaller than the sum of the radii of silica-oxygen ions. This indicates that the silica-oxygen bond has a covalent component and is not purely ionic. Thus, the bonding of silica-oxygen tetrahedra is relatively strong. When Ca^2+^ ions are added to the system, single silicate ions or polymers of multiple silicate ions combine with Ca^2+^ ions to form a large number of C-S-H gels. However, complex salt minerals containing heavy metals and those resembling calcite can cause the disconnection of aluminum–oxygen tetrahedral links from silica-oxygen tetrahedral links, as shown by experimental results [21]. Figure 6 shows that BFS provided Ca^2+^ and Al^3+^ ions, SS provided divalent metal cations and OH- ions, and DG provided Ca^2+^ and SO_4_^2−^. The hydration reaction occurred on the tailings surface and slag micropowder, resulting in the formation of highly insoluble ettringite and driving the reaction to continue. The alumina-like complex salt minerals and calcite-like complicated salt minerals containing heavy metals raise the competition between Al^3+^ and silica-oxygen tetrahedra, leading to a disconnection in the slag. This process brings the reaction back to the hydration reaction centered on the tailings surface and slag micropowder, whilst also promoting the depolymerization of siloxo-tetrahedra and alumino-tetrahedra in BFS and SS.

The effect of multiple raw materials providing each other with key reaction driving factors to promote the continuation of the reaction is called synergism. Using the synergistic effect of SS micropowder, DG micropowder and BFS micropowder, a large number of amorphous C-S-H gels and zeolite-like phases were formed together with a large number of needle-and-rod chalcocite complicated salt crystals, and the needle-and-rod chalcocite complicated salt crystals were tightly enveloped to greatly improve the stability of the whole system [22].

### 2.6. Mechanism of Immobilizing Heavy Metals by SSSDG Solid Waste-Based Cementitious Materials

Through the analysis of the contents of Section 2.5, it is known that the SSSDG solid waste-based gelling material immobilization of the heavy metal mechanism has several aspects. The calcium silicate solid waste-based barrier material hydration process forms C-S-H and other gels, producing aggregate structures that are dense and of extremely low permeability, and the use of hydration products’ metal ions which are bound to them results in them not being easy to leach. Due to the crystal particles of the calcium silica solid waste-based barrier material hydration product being very small, it has a large surface area, so a large number of particles between the micropores can also lead to a large number of metal ions being adsorbed. SSSDG fixed-waste-based gelling materials, through the combination of ionic groups, leads to the formation of very low-solubility complicated salt precipitation, breaking the balance of silica-aluminum in the solution, thereby accelerating the dissolution of aluminum-containing silicates, immobilizing heavy metals while promoting the hydration process [23]. For example, metal Pb^2+^, Al^2+^, Si^4+^, Fe^2+^, Ca^2+^ ions, etc., could be involved in the process of calcite formation, resulting in the formation of lead-containing calcite, thus immobilizing Pb^2+^. The heavy metal ions undergo a complex decomposition reaction in the micropores of the cured material due to the highly alkaline environment provided by the hydration reaction. This results in the formation of low-solubility complex salt precipitates, achieving the desired immobilization effect. The hydration products of SSSDG solid waste-based gelling materials consist of C-S-H gel and calcite, which are silicates. During the process of depolymerization, migration, and re-polymerization, the silica-oxygen tetrahedra can incorporate +3-valent or +5-valent ions, such as Al^3+^, into their network structure. This results in the formation of tetrahedra with four oxygen coordination sites, which are connected to the silica-oxygen tetrahedra at their top angles. Divalent heavy metal ions, such as lead and cadmium, can be trapped in the interstitial space of the network body to balance the charge or replace the trapped Ca ions in its lattice, resulting in firm binding and stabilization. This process prevents the heavy metal ions from escaping into the environment and causing harm [24].

Table 3 shows the conclusions of the mechanisms of heavy metal immobilization in tailings studied by other scholars. It could be found that all of them used the curing method containing calcite, which had the property of being extremely difficult to dissolve under alkaline conditions, which helped to improve the curing effect, compared with the present study. Both involved the formation of C-S-H gels, which had a compact structure and could effectively bind heavy metal ions and reduce their leaching risk. All utilized the adsorption of heavy metal ions by silicate minerals, which were firmly bound and stabilized through the depolymerization and repolymerization processes of silica-oxygen tetrahedra and aluminum–oxygen tetrahedra. However, this study used a variety of curing wastes as the main raw materials, while the materials of the previous studies in Table 3 were generally single curing wastes or non-curing materials. And, this study initially explored the synergistic mechanism of gelling materials and the role of curing heavy metal tailings, while the previous studies in Table 3 focused more on summarizing the different curing mechanisms and their effects on the curing effect of heavy metals.

## 3. Materials and Methods

### 3.1. Materials

The LZT was obtained from the tailings of a lead–zinc mine owned by the Tongling Nonferrous Copper Crown Chizhou Company (Chizhou city, Anhui province, China). It has a content of 17.32% at 200 mesh and is the residual material left after extracting the metal elements from lead–zinc ores. The cement was purchased from the Conch Group. The compressive strengths of the cement were measured using the ISO method (Test method of cement mortar strength, GB/T 17671-2021) [29] after 7 and 28 days, resulting in 39.7 MPa and 51.2 MPa, respectively. These values meet the required cement strength specifications. The sand used in the experiment was ISO standard sand (GSB08-1337).

The gelling material used in this paper was BFS (BFS here stands for blast furnace slag, as explained below the abstract. The main components were SiO_2_ and CaO. Regarding SS, the main chemical components were CaO, Fe_2_O_3_, SiO_2_, MgO, MnO, Al_2_O_3_ and P_2_O_5,_ and DG was the main raw material. BFS was a kind of by-product of iron and steel mills when smelting pig iron. During blast furnace ironmaking, iron ore and fuels (such as coke) were added to the blast furnace. Appropriate amounts of cosolvents, such as limestone and dolomite, were also added to lower the smelting temperature. When the furnace temperature reached 1400~1600 °C, the cosolvents and the iron ore at high temperature produce pig iron and BFS. SS is a steel mill used in the steelmaking process, at a high temperature of 1600 °C or more, through the oxidation of BFS to remove the carbon, silicon, sulfur, phosphorus, manganese and other impurities in the steel produced by the waste BFS. SS mainly comprises oxides resulting from steel oxidation, slagging materials such as silica, fluorite, dolomite, and limestone, impurities in furnace filler materials such as magnesium oxide, the erosion of furnace and lining materials, and alloying materials for adjusting steel properties. DG is a type of sulfate that reacts with Ca(OH)_2_ and aluminum-containing phases produced during cement hydration to form calcium alunite crystals. Calcite crystals grow within the cementitious material and continuously fill the pores, improving the material’s density and mechanical properties.

### 3.2. Methods

#### 3.2.1. Effect of Mechanical Activation on Gelling Activity

An XQM-4 planetary ball mill was used for the test. The LZT was placed in a constant temperature furnace and dried at 100 °C until the moisture content was less than 1%, and then the dried samples were ground at 0.5 h, 1.0 h, 1.5 h, 2.0 h, 2.5 h, 3.0 h, and 3.5 h in a ball mill at a speed of 180 r/min to produce LZT of different fineness levels, according to the “Technical specification for the application of mineral admixture” (GB/T51003-2014) in the strength and activity index test method. The following adulterant accounted for 30% of the total mass of the cementitious system to replace part of the cement. The preparation of cemented sand specimens in standard maintenance conditions (at a temperature of 20 ± 1 °C and a relative humidity not less than 95%) required maintenance for 24 h after demolding, and demolding continued to be maintained in the standard maintenance conditions until the point where the compressive strength test was then carried out. The duration of the maintenance lasted for 3 d, 7 d, and 28 d. The strength test of the collodion specimen was conducted in accordance with the standard for “Standard for test mehod of performance on ordinary fresh concrete” (GB/T 50080-2016) [30]. The specimen size was 70.7 mm × 70.7 mm × 70.7 mm, and each test group prepared three specimens to test the compressive strength of immobilizing for 3 d, 7 d and 28 d, respectively, and the compressive strength test was carried out by using a concrete pressure testing machine.

#### 3.2.2. Preparation of the Immobilization Samples

The immobilizing slurry was formulated by adding water, dry tailings, and cementitious materials in proportion, with a gum/sand ratio of 1:8 and a tailing sand concentration of 65%. The slurry was mixed uniformly and poured into 70.7 mm × 70.7 mm × 70.7 mm triple test molds. The test process was carried out in a constant temperature and humidity box (temperature 20 °C, relative humidity of 92%) while using a gelling agent according to BFS: SS: DG = 80:10:10. After one day of immobilized molding and demolding, the test piece was placed in a sealed plastic box and returned to the constant temperature and humidity box for maintenance. The immobilization process was tested for 3, 7, and 28 days, and the synergistic effect of the SSSDG system on the hydration process was investigated.

#### 3.2.3. Calculation Formula

The gas adsorption method is commonly used to calculate the specific surface area of lead and zinc tailings powder. This method is dependent on factors such as the size and shape of powder particles, surface roughness, and structure. Generally, the specific surface area increases with decreasing particle size, irregular particle shape, rougher surface, and more complex structure.

(1) $$S=\left(V_{m}óN\right)/\left(V_{0}m\right)$$

where S is the micropowder mass specific surface area (m^2^/g); ó is the cross-sectional area of adsorbed gas molecules (nitrogen was used in this study, and the cross-sectional area of its molecules is 0.162 nm^2^. m^2^); N is the Avogadro constant (6.0228 × 10^23^ mol); V_0_ is the volume of 1 mol of adsorbed gas in the standard state, 22.414 × 10^3^ cm^3^; m is the mass of the adsorbed gas (g); and V is the volume of gas in the sample tube (cm^3^/mol).

This formula is obtained by graphing a straight line from the slope and intercept of the straight line to find out the monolayer adsorption of the sample, so as to calculate the specific surface area of the tailings micropowder.

#### 3.2.4. Mineralogical Characterization

X-ray diffraction (XRD) is a very important analytical technique in the field of material science, which reveals the microstructure of a material through the interaction of X-rays with the material. The ARL EQUINOX 1000 X-ray diffractometer was used in this study. The procedure is to grind the solid particles to below 360 mesh using a ball mill and select appropriate X-rays targeted according to the nature of the sample to minimize the effect of fluorescence absorption on the data quality. For the steps of collecting intensity data of diffraction images using an X-ray detector, a background correction and the application of Bragg’s equation to calculate crystal plane spacing, crystal structure resolution and structure refinement are carried out.

The SEM uses a focused electron beam to perform a raster-like scanning motion across the surface of a material to perform imaging and analysis functions based on different types of signals produced by the interaction of the incident electron beam with the sample. It is the most widely used characterization technique in material science. EDS is designed to perform qualitative and semi-quantitative analysis of material elements by detecting the characteristic X-rays excited by the electron beam and the material and can realize the functions of point scanning, line scanning and surface analysis. The model of SEM-EDS used in this experiment is a Zeiss GeminiSEM 450 + Oxford WItec, with the following parameters: secondary electron image resolution: 0.7 nm @ 15 kV and 1.1 nm @ 1 kV; analytical resolution: 2.0 nm @ 15 kV (≥5 nA); magnification: 20~2,000,000×; acceleration voltage: 0.02~30 kV; and spectrometer: resolution (Mn Ka) 127 eV and analytical element range Be4~Cf98.

Specific surface area is an important parameter for measuring the properties of materials, particularly porous or powdered materials. It is closely related to particle size and shape, surface defects and pore structure and can therefore be used as a basis for understanding these physical properties. The size of the specific surface area is directly related to catalytic efficiency. A large surface area means more active sites, which can increase the rate and efficiency of the catalytic reaction. A specific surface analyzer from Kantar (USA) is used in this experiment. Its model is the QuantachromeAutosorb iQ3M. The main parameters are as follows: the instrument is equipped with four degassing stations and three analyzing stations simultaneously, allowing the simultaneous testing of three samples (two microporous or three mesoporous analyses can be performed simultaneously).

## 4. Conclusions

In the present study, through mechanical activation and hydration, it was found that the filling cementitious material using industrial solid wastes such as BFS, SS, and DG as raw materials had a low cost and a superior performance. The material could effectively solidify the heavy metals in LZT and form structural strength-enhancing chalcocite crystals. Experiments showed the best activity of the ball-milled two-hour tailings powder, and the 3- and 7-day compressive strengths of the corresponding specimens were increased by 46.5% and 39.2%, respectively, compared with that of the reference cement. SEM observations showed that calcite interlocked with the C-S-H gel and enhanced the densification. It was shown that the generation of calomel has a significant driving effect on the hydration reaction, which promotes the formation of an insoluble calcite and provides a new approach for the harmless and resourceful treatment of LZT.

## Figures and Tables

**Table 1:** X-ray fluorescence analysis results (wt.%).

Ingredient	SiO_2_	CaO	MgO	Fe_2_O_3_	Al_2_O_3_	Zn	TiO_2_	PO_3_	Pb	Else
Percentage by weight	29.07	28.93	16.69	7.55	7.30	0.24	1.75	0.18	0.21	8.08

**Table 2:** Compressive strength of standard micaceous sand specimens with different ball milling time and immobilization time.

Grinding Time (h)	Compressive Strength (MPa)		
	3 d	7 d	28 d
0.00	10.00	17.40	19.10
0.50	14.00	20.40	22.30
1.00	14.80	21.30	23.10
1.50	16.50	20.90	25.00
2.00	16.60	24.30	26.60
2.50	14.60	21.10	23.20
3.00	13.50	20.70	22.40
3.50	13.00	19.10	22.60
SD (Standard Deviation)	1.97	1.93	2.04
COV (Coefficient of Variation)	13.97%	9.35%	8.85%

**Table 3:** Immobilization mechanism of heavy metals in tailings in different studies.

Al and Si could interact with Pb to form PbAl_2_Si_2_O_8_, thus promoting the immobilization of Pb^2+^. Excess silica and alumina made it difficult to form a stable skeleton, resulting in easy leaching of Pb^2+^. The presence of Zn^2+^ was generally due to the presence of acid-soluble Zn_2_SiO_4_ in the bricks. Zn^2+^ was stabilized by the introduction of Al^3+^ as a host to be replaced by Zn^2+^ in an aluminate or alumino-silicate matrix.	Li et al. (2017) [25]
Calcium carbonate was mainly precipitated in the common crystalline forms of aragonite, calcite and spherulitic aragonite, which have strong bonding properties to connect the lead and zinc tailings particles together. Meanwhile, CO_3_^2-^ and alkalinity caused the heavy metal ions released from the LZT to precipitate mainly in the form of metal carbonates.	Dong et al. (2023) [26]
Hornblende, magnesite, and Pb[Fe_3_(SO_4_)_2_(OH)_6_]_2_ were formed during the hydration process. In addition, calcium silicate hydrate gels and calcite appeared in the cured samples to a high degree of cementation as the duration of hydration increased.	Zhao et al. (2022) [27]
Calcium alunite, C-S-H gels and boron alunite were the main hydration products that immobilized Pb^2+^, Ca^2+^ and Cd^2+^, and these hydration products provided the source of strength.	Wang et al. (2022) [28]

## Data Availability

Data are contained within the article.

## Abbreviations

lead and zinc tailings (LZT), slag (S), steel slag (SS), desulfurization gypsum (DG), slag-steel slag-desulfurization gypsum (SSSDG)
